# Interleukin-6 gene -572G/C polymorphism and prostate cancer risk

**DOI:** 10.4314/ahs.v18i2.10

**Published:** 2018-06

**Authors:** Yingwei Wang, Xin Chen, Yafei Chen

**Affiliations:** Clinical laboratory, Tiantai people's hospital, Tiantai, Zhejiang 317200, China

**Keywords:** Prostate cancer, IL-6 polymorphisms, risk

## Abstract

**Background:**

The aim of the present study was to determine whether the interleukin-6 (IL-6) -572G/C polymorphism correlates with prostate cancer.

**Methods:**

According to inclusion and exclusion criteria, the association of the IL-6 -572G/C polymorphism with prostate cancer was searched in databases and analyzed using comprehensive meta-analysis software. Odds ratios (ORs) with 95% confidence intervals (CIs) were used to assess the strength of the associations.

**Results:**

We considered a total of six case-control studies including 2237 patients and 1754 controls and the meta-analysis results showed significant association between the IL-6 -572G/C polymorphism and prostate cancer risk(CC vs GG: OR = 0.49, 95% CI =0.37–0.65;CG vs GG: OR =0.71, 95% CI = 0.58–0.87; the dominant model: OR =0.65, 95% CI = 0.54–0.79;the recessive model: OR =0.70, 95% CI = 0.58–0.85). In stratified analyses by ethnicity, significant associations were found among Asian populations. However, no significant association was found in Caucasian populations.

**Conclusion:**

Our findings demonstrated that the -572G/C polymorphism of the IL-6 gene may be a risk factor for the development of prostate cancer in Asians.

## Introduction

Prostate cancer is a common cause of cancer-related death and is one of the most commonly diagnosed malignancies in men^1^. Recently numerous medical studies have made significant progress in the field. Age, family history, and race are the most consistently observed risk factors associated with prostate cancer. Especially age and inherited factors are estimated to be responsible for 5% to 9% percentage of this disease^2^. Recently, molecular biological and epidemiological studies results suggest that the pathogenesis of prostate cancer may be associated with single nucleotide polymorphisms (SNPs) among several genes^3^–^4^.

Interleukin-6(IL-6) is a pleiotropic cytokine that not only exhibits immunological effect but also functions in haematopoiesis, bone metabolism, and tissue regeneration^5^. In addition, IL-6 is widely believed to play a key role in the pathogenesis of many cancers^6^. Located on chromosome 7p21, the IL-6 gene is composed of five exons, four introns and a promoter region^7^. Many studies have focused on the promoter region of the IL-6 gene for many polymorphisms are identified in this region. Functional polymorphism(−572G/C, rs1800796) in the promoter region is associated with increased plasma levels of IL-6, which has been reported to influence IL-6 levels^8^. A previous meta-analysis suggested that the IL6-174G/C polymorphism is associated with an increased risk of prostate cancer^9^.

Several studies have been performed to figure out whether there is an association between prostate cancer and the IL-6 −572G/C polymorphism, but the results are equivocal. Meta-analysis combines the results from relevant studies, explores the heterogeneity and identifies subgroups associated with the factor of interest.Therefore, we performed this meta-analysis to clarify the association of this variant with prostate cancer.

## Methods

### Literature search and selection criteria

PubMed, Embase, Web of Science, Google Scholar, China Biological Medicine Database (CBMD), and the China National Knowledge Infrastructure (CNKI) databases were searched for papers linking the IL6 −572G/C polymorphism prior to March 2017, using the keyword: “interleukin 6/IL6”,“−572G/C”,,“prostate cancer”,“polymorphism”. The search was not limited to English language articles. Furthermore, the reference lists of retrieved articles were manually screened to obtain relevant papers.

### Criteria for inclusion and exclusion

Studies included in this meta-analysis meet the following inclusion criteria: I) case-control design; II) evaluation of the correlation of IL-6 −572G/C polymorphism with prostate cancer risk; and III) adequate data to calculate the odds ratio (OR) and its corresponding 95% confidence interval (CI). Of studies published, the same case series, we selected the most recent papers. In addition, studies without effective information were excluded after the efforts to extract data from the included paper.

### Data extraction

The following information was extracted: a) authors, b) year of publication, c) country, d) ethnicity of the study subjects, e) sample size, f) allele and genotype distribution, and g) evidence of Hardy-Weinberg equilibrium (HWE) in the control population.

### Statistical analysis

Meta-analysis was performed using a comprehensive meta-analysis software program (BiostatCorporation, NJ, USA).The pooled OR and 95% CI were used to investigate the association between the risk of prostate cancer and IL-6 −572G/C polymorphism. In the present meta-analysis, we adopted the following genetic models:a homozygote comparison(CC vs GG), a heterozygote comparison(CG vs GG), a dominant model(CC+CG vs GG) and a recessive model (CC vs CG+GG)^10^. The variation caused by heterogeneity was estimated by calculating the inconsistency index I2, the random effects model was used to calculate the OR and 95% CI when I2>50%. Otherwise, the fixed effect model was applied. Publication bias was evaluated by Egger's regression. A P value less than 0.05 was considered statistically significant.

## Results

### Study characteristics

**Figure 1:**
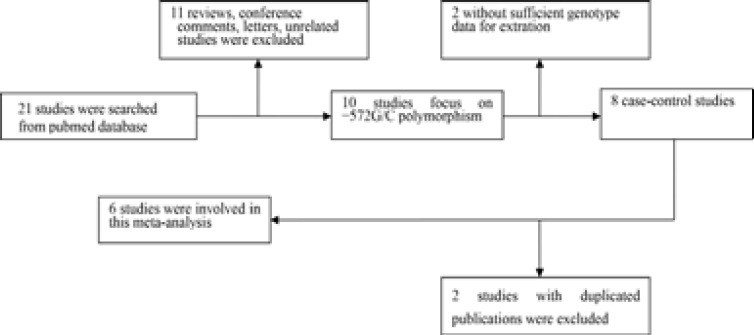
Study selection and inclusion process.

The search strategy retrieved 21 relevant papers. Based on the inclusion criteria, 6 case-control studies met inclusive criteria 11–16, and 15 studies were excluded. The flow chart for the study selection is shown in Figure 1. These 6 papers selected included 2237 cases and 1754 healthy controls. The HWE test was performed on genotype distribution of the controls, all included studies were in HWE (P>0.05) except Bao et al.The baseline characteristics of included studies are summarized in Table 1.

**Table 1:** Included studies of the Interleukin-6 gene -572G/C polymorphism with prostate cancer

First author/year	Country	Ethnicity	Cases/Controls	−572G/C (Case/control)			HWE test

				CC	CG	GG	
Sun et al 2004	Sweden	Caucasian	1337/753	2/4	109/74	1226/675	0.21
Bao et al 2008	China	Asian	136/120	50/65	39/27	47/28	0.00
Wang et al 2009	USA	Caucasian	116/109	1/0	19/25	233/225	0.41
Lu et al 2011	China	Asian	200/279	97/150	60/100	43/29	0.05
Chen et al 2015	China	Asian	212/237	79/110	96/102	37/25	0.85
Huang et al 2016	China	Asian	236/256	117/144	88/89	31/23	0.09

HWE, Hardy-Weinberg equilibrium

### Meta-analysis results

**Figure 2:**
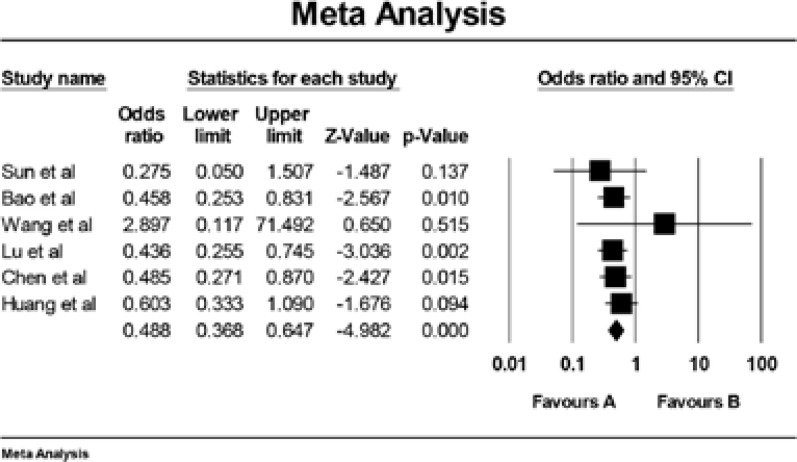
Meta-analysis of the relationship between the IL-6 −572G/C polymorphism and prostate cancer risk(CC vs GG).

The combined data showed that the IL-6 −572G/C polymorphism is associated with increased prostate cancer risk in various genetic models indicated in Table 2 (CC vs GG: OR = 0.49, 95% CI =0.37–0.65, Figure 2; CG vs GG: OR =0.71, 95% CI = 0.58–0.87; the dominant model: OR =0.65, 95% CI = 0.54–0.79; the recessive model: OR =0.70, 95% CI = 0.58–0.85). When stratified according to ethnicity, we detected a significant association in Asians, but not in Caucasians.

**Table 2:** Summary ORs and 95%CI of Interleukin-6 gene -572G/C polymorphism with prostate cancer risk

Variables	N a	CC vs GG		CG vs GG		Dominant model		Recessive model	

		OR(95%CI)	Model	OR(95%CI)	Model	OR(95%CI)	Model	OR(95%CI)	Model
**Total**	6	0.49(0.37–0.65)	F	0.71(0.58–0.87)	F	0.65(0.54–0.79)	F	0.70(0.58–0.85)	F
**Ethnicity**									
Asian	4	0.49(0.37–0.65)	F	0.62(0.45–0.83)	F	0.54(0.41–0.71)	F	0.71(0.58–0.86)	F
Caucasian	2	0.46(0.10–2.07)	F	0.80(0.60–1.05)	F	0.78(0.60–1.03)	F	0.47(0.11–2.12)	F
**HWE**									
yes	5	0.50(0.36–0.68)	F	0.69(0.56–0.86)	F	0.66(0.54–0.81)	F	0.75(0.61–0.92)	F
**no**	1	0.46(0.25–0.83)	/	0.86(0.44–1.70)	/	0.58(0.33–1.00)	/	0.49(0.30–0.81)	/

a Number of comparisons.

**Figure 3:**
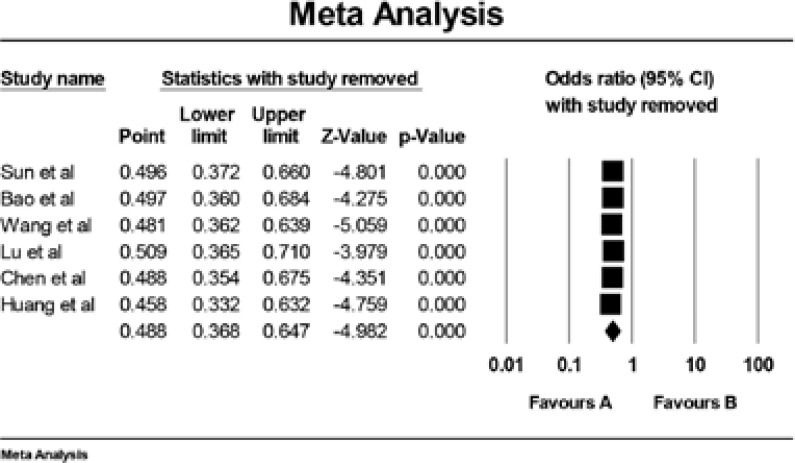
Sensitivity analysis of the IL-6 −572G/C polymorphism with prostate cancer risk.

When patients were stratified according to HWE, a significant association was found in the studies consistent with HWE. Sensitivity analysis was performed via assess the influence of each individual paper on the pooled OR via deleting one single study each time.There is no single article that influenced the pooled ORs, suggesting that the results are stable(Figure 3).

### Publication bias

**Figure 4:**
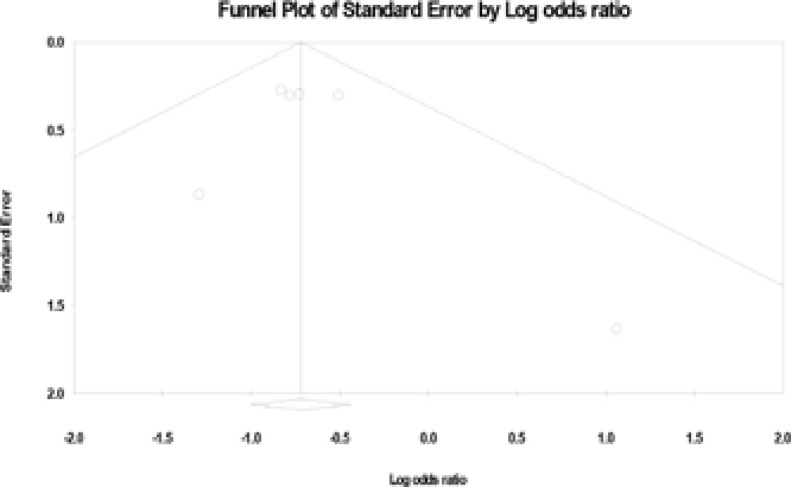
Egger's funnel plot test.

The funnel plot was performed to assess the publication bias. There was no evidence of publication bias visually from the funnel plot (Figure 4), which showed that the publication bias of our meta-analysis was low.

## Discussion

Prostate cancer is the second most common cancer diagnosed in men, and the fourth most common cancer diagnosed worldwide. Although genetic variants appear to play an important role in prostate cancer risk, the precise mechanism is likely to be complex^11^. Recombinant adenovirus-p53 (rAd-p53) (Gendicine) is a newly developed medicine of gene therapy that relies on the function of wild-type P53, which is safe and could prolong the survival time of the patients with hepatocellular carcinoma and nasopharyngeal carcinoma^12^,^13^. There have been no reports regarding prostate cancer to date. As a multifactorial cytokine, it is generally accepted that IL-6 plays a significant role in the pathogenesis of many forms of cancer^6^. Several studies have shown an association between the IL-6 gene −572G/C polymorphism and the risk of prostate cancer, but the results are inconclusive. In order to provide comprehensive and reliable conclusions, we performed the present meta-analysis comprising six independent case-control studies, including 2237 cases and 1754 controls.

From the combined meta-analysis results, the data suggest sthat −572G/C polymorphism was associated with the risk of prostate cancer. For the difference in the environment in which they lived in, we perform a ethnicity-specific subgroup analysis, and significant association was found in Asians, but not in Caucasians. Deviation of allelic distributions from HWE may contribute to between-study heterogeneity, the sub-group analysis via limiting this meta-analysis to those papers that are consistent with HWE revealed that our data was robust. No evidence showed publication bias in this meta-analysis.

The meta-analysis suggested that the GG genotype of IL-6 −572G/C polymorphism might be related to the increased risk of prostate cancer in Asians. Nevertheless, the mechanism of how this polymorphism relates to prostate cancer risk remains unclear. Previous study suggested that the GG and GC genotypes of the −572G/C polymorphism are associated with higher IL-6 serum levels than in the CC genotype^14^. An elevated serum IL-6 level has been correlated with prostate cancer risk^15^. In addition, it is common to find discordant results between Asians and Caucasians with respect to gene polymorphisms, partly owing to a possible role of ethnic differences in genetic backgrounds and the environment in which they lived^16^.

Although our study provides a better understanding of the IL-6 −572G/C polymorphism and susceptibility to prostate cancer, it has limitations. Firstly, only two studies involving Caucasian patients met inclusion criteria. This may not provide enough statistical power to detect the possible effects of the −572G/C polymorphism in Caucasian patients. Secondly, original individual data could not be extracted from each study and our results were based on unadjusted estimates, the introduction of heterogeneity is therefore unavoidable and may affect our results. Thirdly, the possibility of gene-gene interactions and environmental factors were also not considered in our study.

In summary, the present meta-analysis indicated that the IL-6 −572G/C polymorphism might increase the prostate cancer risk in the Asian population.
